# Deep learning and inflammatory markers predict early response to immunotherapy in unresectable NSCLC: A multicenter study

**DOI:** 10.17305/bb.2025.12324

**Published:** 2025-06-10

**Authors:** Lei Yuan, Qi Wang, Fei Sun, Hongcan Shi

**Affiliations:** 1 Department of Thoracic Surgery, Northern Jiangsu People’s Hospital Affiliated to Yangzhou University, Yangzhou, China; 2 Institute of Translational Medicine, Medical College, Yangzhou University, Yangzhou, China; 3 Department of Thoracic Surgery, The Affiliated Taizhou People’s Hospital of Nanjing Medical University, Taizhou, China

**Keywords:** Artificial intelligence, deep learning, non-small cell lung cancer, NSCLC, inflammatory parameter, immunotherapy

## Abstract

Immune checkpoint inhibitors (ICIs) demonstrate substantial interpatient variability in clinical efficacy for unresectable non-small cell lung cancer (NSCLC), underscoring the unmet need for noninvasive biomarkers to predict early therapeutic responses and improve survival outcomes. To address this, we developed a computed tomography (CT)-based deep learning model integrated with the systemic immune-inflammatory-nutritional index (SIINI) for early prediction of ICI response. In a retrospective multicenter study of 265 patients treated with ICIs (incorporating chest CT and laboratory data), the cohort was divided into training (70%), internal validation (30%), and external validation sets. The combined model—leveraging DenseNet121-derived deep radiomic features alongside SIINI—achieved strong predictive performance, with AUCs of 0.865 (95% CI: 0.7709–0.9595) in the internal validation cohort and 0.823 (95% CI: 0.6627–0.9827) in the external validation cohort. Gradient-weighted class activation mapping highlighted key CT regions contributing to model predictions, enhancing interpretability for clinical application. These findings highlight the potential of integrating deep learning with inflammatory biomarkers to support personalized ICI therapy in unresectable NSCLC. Future directions include incorporating multi-omics biomarkers, expanding multicenter validation, and increasing sample sizes to further improve predictive accuracy and facilitate clinical translation.

## Introduction

Lung cancer remains a leading type of cancer and the foremost cause of cancer fatalities worldwide [1, 2]. Non-small cell lung cancer (NSCLC) accounts for the majority of lung cancer cases (80%–90%) and is often diagnosed at advanced stages (65%), frequently presenting with local or distant metastases [3], which often precludes surgical intervention. Recent advancements in immunotherapy, particularly the application of immune checkpoint inhibitors (ICIs), have shown significant promise in improving outcomes for patients with unresectable NSCLC [4]. However, the variable response to immunotherapy highlights the need for further investigation into predictive biomarkers that can forecast immune response. Accumulating evidence has implicated various biomarkers in predicting responsiveness to ICIs in NSCLC, including tumor mutational burden (TMB) [5], programmed death ligand-1 (PD-L1) expression [6], tumor-infiltrating lymphocyte (TIL) density [7], and inflammatory cytokine profiles [8]. Current biomarker assessment protocols predominantly rely on invasive tissue biopsies, which present dual clinical challenges: procedure-related morbidity risks and limited capacity to map intratumoral heterogeneity due to inherent sampling constraints [9, 10]. This critical methodological gap necessitates the development of robust non-invasive biomarkers capable of predicting therapeutic outcomes in patients with unresectable NSCLC undergoing ICI regimens.

Emerging evidence underscores the intricate interplay between tumor pathogenesis and host inflammatory response, immune status, and nutritional profile [11–15]. The systemic immune-inflammation-nutritional index (SIINI) is an innovative multidimensional biomarker that combines pre-treatment inflammatory indicators [16], immunocompetence metrics, and nutritional determinants, theoretically providing a more comprehensive evaluation of pretherapeutic host status compared to conventional unidimensional biomarkers. Nevertheless, the prognostic utility of SIINI in predicting clinical outcomes for NSCLC patients receiving ICIs remains unexplored.

Beyond conventional laboratory diagnostics, computed tomography (CT)-based imaging biomarkers have become indispensable in the diagnostic workflow of lung cancer [17]. The integration of artificial intelligence with medical imaging has catalyzed the emergence of radiomics-driven deep learning (RDL) in thoracic oncology, enabling the quantitative extraction of high-dimensional imaging features that are imperceptible to human visual assessment [18]. This computational approach facilitates the development of non-invasive predictive signatures for diverse clinical applications, including tumor characterization [19], therapeutic strategy optimization [20], and treatment response monitoring. Notably, foundational studies have established the prognostic relevance of conventional radiomic features in both localized and advanced NSCLC. For resectable disease, radiomic signatures demonstrate predictive capacity for neoadjuvant chemotherapy response [21], while in advanced stages, specific imaging biomarkers correlate with immunotherapy outcomes [22]. These findings underscore the evolving role of quantitative imaging biomarkers in precision oncology paradigms. However, there is currently limited evidence to substantiate the effectiveness of integrating clinical data, particularly systemic immune-inflammatory-nutritional indexes such as SIINI, into deep learning models to predict the response of patients with unresectable NSCLC toICIs. Moreover, CT-based RDL can reveal heterogeneity within the tumor and provide a potential research direction for multidimensional interpretation of the tumor microenvironment [23, 24].

In this study, we aimed to investigate the early predictive capability of a CT-based deep learning model combined with the inflammation parameter SIINI for predicting the response of unresectable NSCLC patients to ICIs, utilizing clinical data from 265 patients across two independent medical centers.

## Materials and methods

### Data collection

In this study, we selected patients with unresectable NSCLC who were treated with single-agent ICIs at Northern Jiangsu People’s Hospital (Center A) and Taizhou People’s Hospital (Center B) (Ethical Review No. 2021ky211; KY 2024-093-01). Patients received either 200 mg of Pembrolizumab every three weeks, 3 mg/kg of Nivolumab every two weeks, or 200 mg of Sintilimab every three weeks. The planned study period is from January 2021 to December 2024. All procedures adhere to the guidelines and ethical principles outlined in the 1964 Declaration of Helsinki.

Inclusion criteria:

(1) Eastern Cooperative Oncology Group (ECOG) performance status of 0–3; (2) Presence of measurable lung lesions as per Response Evaluation Criteria in Solid Tumors (RECIST V1.1), as determined by standard chest CT scans; (3) Diagnosis of NSCLC confirmed by biopsy or bronchofibroscopy and histopathological examination, with staging based on imaging and pathology according to the TNM (8th edition) classification as stage IIIB to IV [25]; (4) The comprehensive availability of laboratory and imaging data for evaluating disease progression includes standard blood work and biochemical analyses carried out before the commencement of ICI therapy, along with chest CT scans performed every 6–8 weeks thereafter; (5) Comprehensive follow-up information available.

Exclusion criteria:

(1) Inadequate image quality, such as presence of artifacts; (2) History of thoracic surgery; (3) Loss to follow-up after receiving immunotherapy; (4) Inability to obtain complete laboratory and imaging data for pathological evaluation.

### Clinical data

We collected baseline data of patients and laboratory test results, including age, gender (female ═ 0/male ═ 1), smoking history (current or former smokers ═ 1/Never smokers ═ 0), basic disease (with basic disease ═ 1/without basic disease ═ 0), body mass index (BMI), treatment lines, medication regimen (Pembrolizumab ═ 1/Nivolumab ═ 2/Sintilimab ═ 3), EGFR mutation (Positive ═ 1/Negative ═ 0), TNM (IIIB ═ 3/IV ═ 4), ECOG, pathological type (adenocarcinoma ═ 1/squamous cell carcinoma ═ 0), modality, PD-L1 expression (No record ═ 0, tumor proportion score, TPS < 1% ═ 1, TPS ≥ 1%–49% ═ 2, TPS ≥ 50% ═ 3), etc.

Blood cell counts encompassed white blood cell enumeration, neutrophil, lymphocyte, monocyte, eosinophil, and basophil quantifications along with their respective percentages. Additionally, hemoglobin concentration, red blood cell count, hematocrit level, platelet count, proportion of larger platelets, and plateletcrit were determined. Blood biochemistry analyses comprised measurements of total protein, albumin, and levels of LDH, ALT, AST, urea, and creatinine.

Based on clinical retrospective data, this study found that neutrophil count, lymphocyte count, platelet count, hemoglobin level, serum albumin level, and BMI before treatment were calculated by neutrophil count × platelet count × hemoglobin level/(lymphocyte count × BMI × serum albumin level) to form a new index—SIINI. At the same time, PNI, PLR, ALI, SII, and NLR calculated from these indicators were also included in the subsequent clinical prediction model studies, and the calculation formulas and data were in the Supplemental data. The full names of some laboratory test results can be found in the abbreviation list at the end of this article.

### Image acquisition

All patients underwent chest CT scans prior to the initiation of ICI therapy. Resultant imagery underwent moderate-detail reconstruction, yielding slice widths of 3–5 mm. Tumor segmentation was subsequently performed to delineate the primary NSCLC lesions. Using 3D-Slicer v4.11, two cancer specialists jointly delineated regions of interest (ROIs) and derived radiomic features from each detected nodule. The target lesion was defined as any tumor mass measuring ≥ 5 millimeters in diameter, which was distinctly marked at baseline and consistently observed in follow-up CT scans. Response assessment based on the follow-up CT scans adhering to RECIST 1.1 criteria categorized patients into responders (label═1) exhibiting complete remission (CR), partial remission (PR), or stable disease (SD), while those with progressive disease (PD) were classified as non-responders (label═0). All CT scan interpretations were conducted by two independent oncologists to ensure objectivity.

To enhance reliability, two researchers (QW and FS) independently outlined the ROIs. Each researcher repeated this process for the same tumor at different time points. Intra-group consistency of the extracted radiomic features was evaluated using the intra-class correlation coefficient (ICC), thereby ensuring robustness in the data collected. After calculating the ICC within and between groups, the characteristics of ICC > 0.8 at both time points were selected. Any differences were resolved through discussions between the two researchers.

### Methods

**Figure 1.**
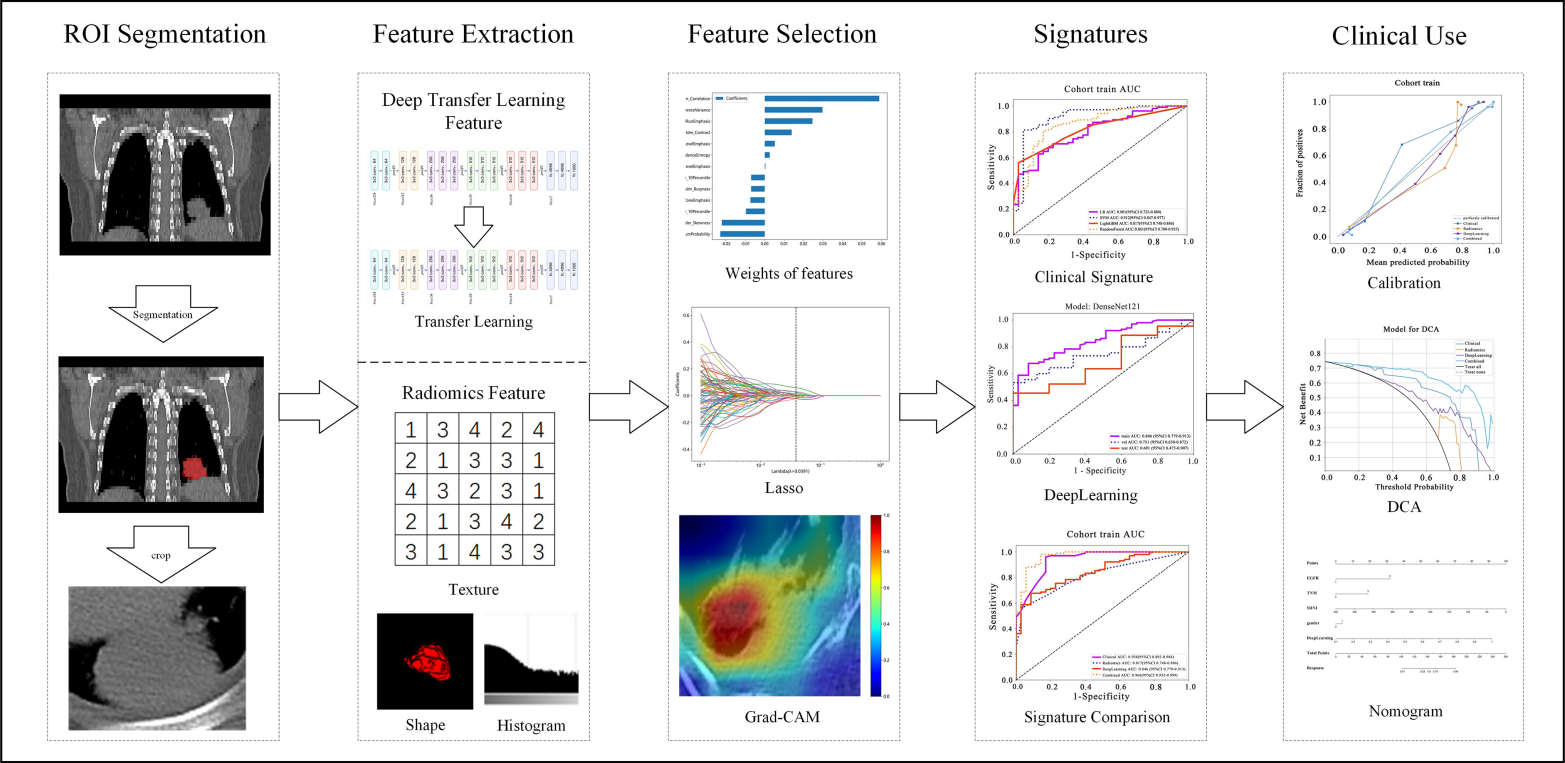
**Workflow of this study.** It show the development process of the deep learning combined with inflammatory markers model, including ROI segmentation, extraction of features, screening, visualization, and model evaluation. ROI: Region of interest; LASSO: Least Absolute Shrinkage and Selection Operator; Grad-CAM: Gradient-weighted class activation mapping; DCA: Decision curve analysis.

**Figure S1.**
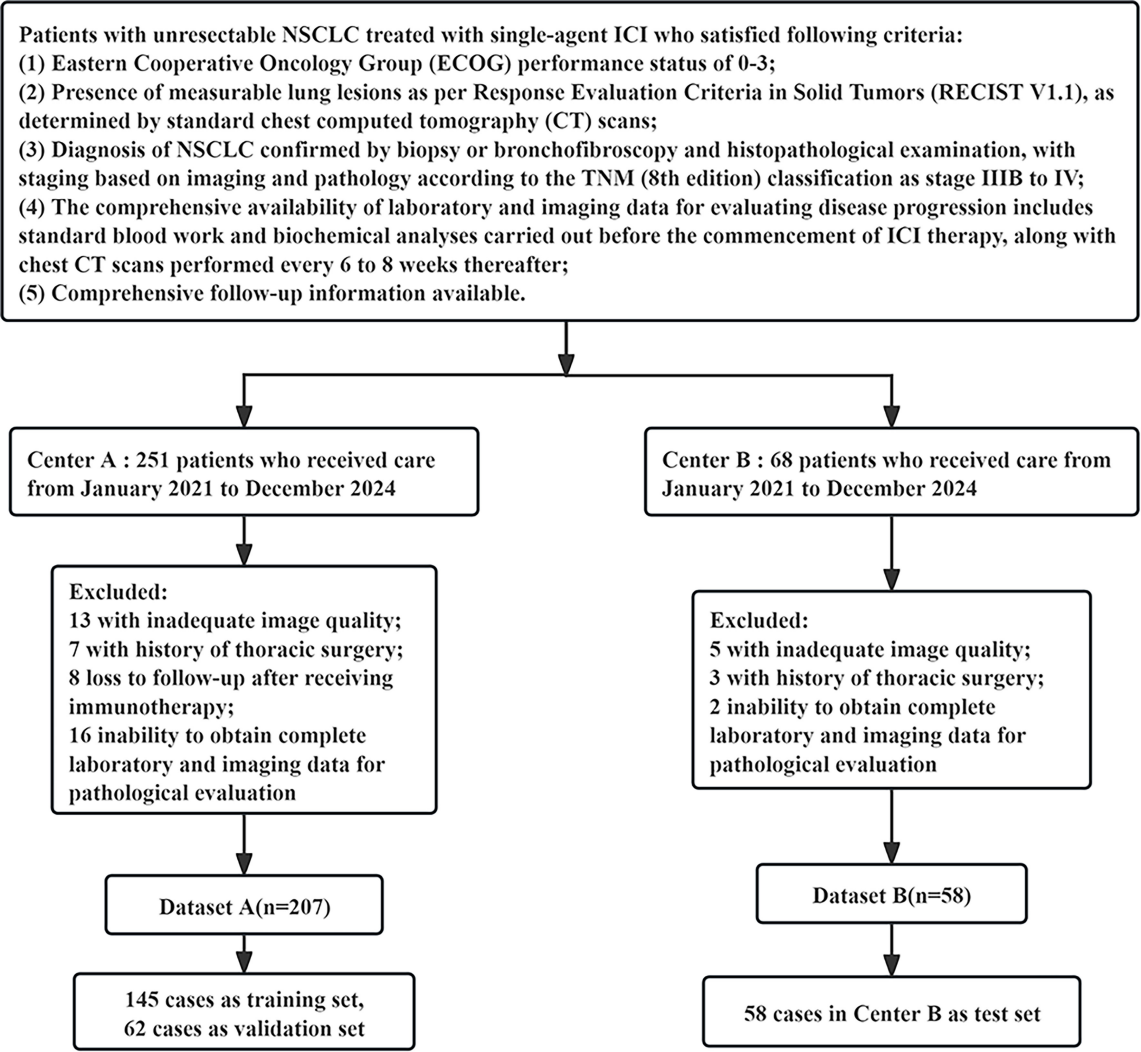
Research flowchart.

We have developed the workflow shown in Figure 1 to carry out this research. This study included 265 participants, comprising 207 patients from Center A; Center B had 58 cases, with 145 cases as the training set, 62 cases as the validation set, and 58 cases in Center B as the test set (Figure S1).

### Data preprocessing

In our medical image analysis, voxel spacing was standardized across all volumes of interest to a uniform resolution using a fixed resolution resampling method. At the same time, CT Hounsfield Units (HU) were limited to a range of −400 to 600. This standardization process was crucial for enabling precise image comparisons, significantly enhancing the accuracy and reliability of our analytical outcomes.

### Radiomics procedure

#### Feature extraction

In this study, we’ve neatly divided radiomic features into three main buckets: (I) Shape and size descriptors, (II) Intensity-based measures, and (III) Texture features. Shape and size descriptors are all about capturing the 3D form of the tumors. Intensity-based measures look at the spread of voxel intensities using basic statistical tools. On the flip side, texture features dig into the patterns and how voxel intensities are arranged in space, using more complex statistical methods like second-order and higher-order analyses.

In analyzing the texture, we utilized well-established methods including the gray-level co-occurrence matrix (GLCM), gray-level run-length matrix (GLRLM), gray-level size zone matrix (GLSZM), and neighborhood gray-tone difference matrix (NGTDM). Each specified subregion underwent feature extraction using the PyRadiomics tool (version 3.0.1), following the protocols established by the Imaging Biomarker Standardization Initiative (IBSI) meticulously.

#### Feature selection

In the feature selection process, we adopted a layered approach. We began by standardizing the features using Z-scores, followed by *t*-tests to assess their significance. Any feature with a *P* value below 0.05 was approved to proceed to the next round. Next, features with high reproducibility were evaluated using Pearson’s correlation coefficient. For pairs of features that exhibited a correlation greater than 0.9, we implemented a strategic recursive elimination process to retain a single representative feature from each highly correlated pair, thereby minimizing redundancy. Finally, we refined the radiomic signature using Least Absolute Shrinkage and Selection Operator (LASSO) regression, which effectively reduced the influence of non-contributory features. The optimal regularization parameter (λ) was determined using 10-fold cross-validation.

#### Radiomics signature

Following feature refinement via LASSO regression, risk evaluation was conducted utilizing both linear models (such as logistic regression [LR]) and tree-based models (including random forest and LightGBM). Model hyperparameter optimization was performed through 10-fold cross-validation within the training dataset, employing the GridSearch algorithm to fine-tune parameters. The parameters demonstrating the highest median efficacy were selected for final model training.

### Deep learning procedure

#### Data preparation

Crop ROI: In our methodology, we identified the slice with the largest ROI for each patient as the representative image. To streamline analysis and minimize interference, the ROI was confined to its minimal bounding rectangle, extended by 10 pixels. This expansion recognizes the significance of peritumoral regions, as highlighted by recent studies [26].

Data augmentation: The intensity distribution across RGB channels for the input images was standardized using Z-score normalization. During the training stage, real-time data augmentation enhanced model resilience through random crops and horizontal/vertical flips. For test images, processing was limited to normalization to maintain consistency.

#### Model training

Transfer Learning: Previous studies have demonstrated that DenseNet121 [27, 28], with its unique dense connection mechanism, significantly outperforms traditional CNN models (such as ResNet and VGG) in feature reuse, parameter efficiency, training stability, and task adaptability. Its advantages are particularly pronounced in scenarios requiring high precision and efficient feature extraction, such as medical imaging and target detection. This study utilized the advanced architecture of DenseNet121 to achieve superior performance compared to traditional CNN-based models. We conducted comparative analyses of these networks to identify the most effective model for our specific research needs.

Hyperparameters: Our strategy incorporated transfer learning to accommodate diverse patient populations and variability. The models were initialized with ImageNet-derived parameters for enhanced adaptability. Our methodology focused on meticulous calibration of the learning rate, employing a cosine decay strategy to maximize generalization across varied datasets.



(1)

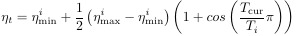




Here, 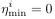 represents the minimum learning rate, 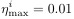 sets the maximum learning rate, and 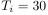 denotes the number of epochs for each training cycle. Additional critical hyperparameters included the use of stochastic gradient descent (SGD) as the optimizer and softmax cross-entropy as the loss function.

#### Deep learning signature

In our model, the probabilities outputted by the DenseNet121 are defined as the deep learning signature, representing the model’s predictive capabilities.

### Clinical use

Clinical signature: We employed the same model used for the radiomics signature to model our clinical task. We then selected the model that performed best on the test set for subsequent comparisons of the signatures. This approach ensured that the most effective predictive model was utilized for clinical evaluation.

Combined model: To enhance its clinical utility, we carried out univariable and stepwise multivariable analyses on all clinical features to identify significant predictors. These selected clinical features were integrated with outputs from our deep learning model to develop an LR linear model, resulting in the formation of the combined signature. We employed a nomogram for effective visualization of this signature.

Metrics: We gauged how well our models could distinguish between true and false positives by using receiver operating characteristic (ROC) curves. To see if our models were well calibrated, we plotted calibration curves and then ran Hosmer-Lemeshow (HL) tests to really put them through their paces. On top of that, we performed decision curve analysis (DCA) to figure out if our predictive models would actually be helpful in a clinical setting.

### Ethical statement

This study was conducted according to the ‘Helsinki Declaration.’ Besides, this study was carefully reviewed by the Ethical Review Committee of Northern Jiangsu People’s Hospital and Taizhou People’s Hospital (Ethical Review No. 2021ky211; No. KY 2024-093-01), which unanimously agreed that the patients’ hospitalization data and images used in this retrospective study were exempted from the informed consent application in the ethics committees and approved by the committees.

### Statistical analysis

We randomly split the dataset, earmarking 70% for training and setting aside the remaining 30% for internal validation. To really put our model through its paces and see how well it generalized, we also tapped into data from an outside center, using it as an external validation set. Table 1 shows the baseline characteristics of this study.

**Table 1:** Baseline characteristics

**Feature_name**	**Train-label ═ 0**	**Train-label ═ 1**	***P* value**	**Val-label ═ 0**	**Val-label ═ 1**	***P* value**
Age	65.97 ± 10.21	67.61 ± 8.34	0.499	66.87 ± 10.22	65.71 ± 10.02	0.702
BMI	22.76 ± 3.75	22.47 ± 3.94	0.618	24.21 ± 4.55	22.67 ± 4.58	0.253
Modality	3.17 ± 1.92	3.11 ± 1.60	0.992	3.07 ± 1.98	3.40 ± 1.74	0.605
NLR	4.83 ± 2.93	3.41 ± 1.01	0.002	3.99 ± 1.61	3.27 ± 0.95	0.298
PLR	202.05 ± 107.29	191.07 ± 94.95	0.784	189.75 ± 75.05	197.81 ± 127.05	0.67
ALI	290.00 ± 236.72	403.32 ± 1086.61	0.717	284.93 ± 141.93	317.74 ± 250.06	0.905
SII	1109.62 ± 924.00	922.70 ± 852.36	0.255	994.24 ± 591.15	794.51 ± 648.57	0.116
PNI	45.73 ± 5.97	46.97 ± 5.65	0.272	47.91 ± 5.24	48.77 ± 5.65	0.604
SIINI	179.80 ± 69.18	120.42 ± 59.06	<0.001	169.75 ± 49.88	123.02 ± 70.43	0.003
*Gender*			0.025			0.835
Female	10 (28.57)	11 (10.78)		3 (20.00)	6 (13.33)	
Male	25 (71.43)	91 (89.22)		12 (80.00)	39 (86.67)	
*Smoking*			0.826			0.343
0	13 (37.14)	42 (41.18)		7 (46.67)	13 (28.89)	
1	22 (62.86)	60 (58.82)		8 (53.33)	32 (71.11)	
*Basic_disease*			0.317			0.536
0	16 (45.71)	35 (34.31)		7 (46.67)	15 (33.33)	
1	19 (54.29)	67 (65.69)		8 (53.33)	30 (66.67)	
*ECOG*			0.095			0.013
0	null	3 (2.94)		null	2 (4.44)	
1	13 (37.14)	58 (56.86)		4 (26.67)	25 (55.56)	
2	15 (42.86)	31 (30.39)		6 (40.00)	16 (35.56)	
3	7 (20.00)	10 (9.80)		5 (33.33)	2 (4.44)	
*EGFR*			<0.001			0.002
0	6 (17.14)	86 (84.31)		7 (46.67)	40 (88.89)	
1	29 (82.86)	16 (15.69)		8 (53.33)	5 (11.11)	
*PD_L1*			0.247			0.339
0	32 (91.43)	78 (76.47)		12 (80.00)	38 (84.44)	
1	2 (5.71)	11 (10.78)		1 (6.67)	5 (11.11)	
2	1 (2.86)	8 (7.84)		1 (6.67)	2 (4.44)	
3	null	5 (4.90)		1 (6.67)	null	
*Medication_regimen*			0.291			0.318
1	13 (37.14)	29 (28.43)		7 (46.67)	13 (28.89)	
2	19 (54.29)	69 (67.65)		8 (53.33)	29 (64.44)	
3	3 (8.57)	4 (3.92)		null	3 (6.67)	
*Treatment_lines*			0.389			0.342
1	20 (57.14)	69 (67.65)		8 (53.33)	28 (62.22)	
2	12 (34.29)	23 (22.55)		6 (40.00)	10 (22.22)	
3	3 (8.57)	10 (9.80)		1 (6.67)	7 (15.56)	
*Type*			0.578			0.732
1	33 (94.29)	100 (98.04)		15 (100.00)	42 (93.33)	
2	2 (5.71)	2 (1.96)		null	3 (6.67)	
*Pathological_type*			0.399			1.0
0	16 (45.71)	57 (55.88)		7 (46.67)	20 (44.44)	
1	19 (54.29)	45 (44.12)		8 (53.33)	25 (55.56)	
*TNM*			0.004			0.408
3	3 (8.57)	37 (36.27)		6 (40.00)	11 (24.44)	
4	32 (91.43)	65 (63.73)		9 (60.00)	34 (75.56)	

**Notes:** Gender (female ═ 0/male ═ 1), smoking history (current or former smokers ═ 1/Never smokers ═ 0), basic disease (with basic disease ═ 1/without basic disease ═ 0), medication regimen (Pembrolizumab ═ 1/ Nivolumab ═ 2/ Sintilimab ═ 3), EGFR mutation (Postive ═ 1/Negative ═ 0), TNM (IIIB ═ 3/IV ═ 4), pathological type (adenocarcinoma ═ 1/squamocellular carcinoma ═ 0), PD-L1 expression (No record ═ 0, TPS <1% ═ 1, TPS≥1%–49% ═ 2, TPS≥50% ═ 3). PD-LI: Programmed death ligand-1; SIINI: Systemic immune-inflammatory-nutritional index.

We ran our analyses using Python 3.7.12 and the statsmodels package, version 0.13.2. When it came to building our machine learning models, we leaned on scikit-learn, specifically version 1.0.2. For the deep learning side of things, we harnessed the power of an NVIDIA 4090 GPU, along with the MONAI (version 0.8.1) and PyTorch (version 1.8.1) frameworks.

## Results

### Clinical features analysis

**Figure 2.**
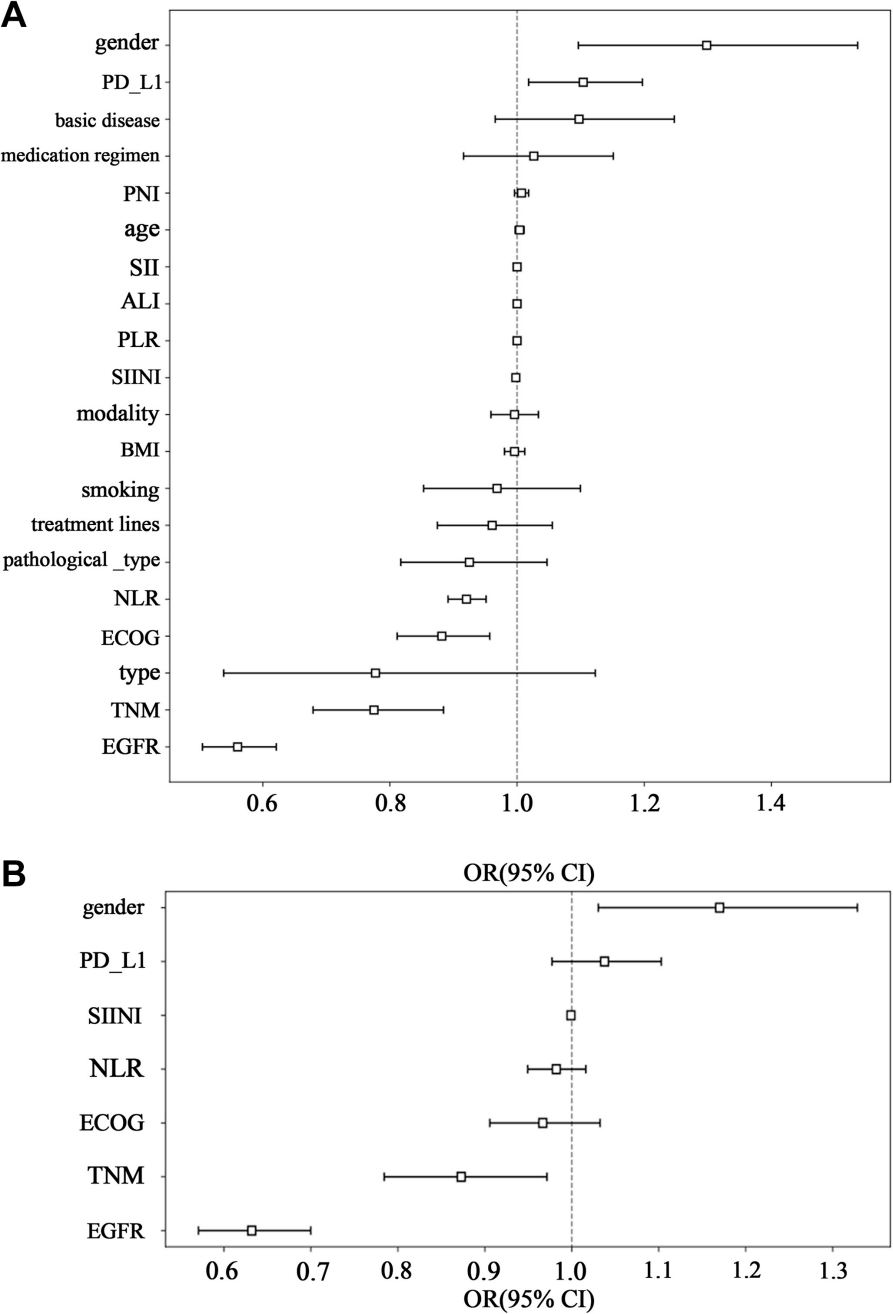
**OR of clinical features: (A) OR of clinical features in univariable analysis; (B) OR of clinical features in multivariable analysis.** CI: Confidence interval; PD-LI: Programmed death ligand-1; SIINI: Systemic immune-inflammatory-nutritional index.

Univariable and multivariable analysis: In our research, we performed an extensive univariate analysis of all clinical features, calculating the OR and associated *P* values for each. Features with a *P* value less than 0.05 were selected for inclusion in the nomogram construction (Figure 2). Additionally, we constructed a clinical model based on these clinical features, including EGFR, TNM, SIINI, and gender. To some extent, these indicators present the body’s nutritional inflammation status and tumor heterogeneity of unresectable NSCLC patients using ICIs, which can be included in the prediction model after multivariate variable analysis (Table S1 and Table S2).

**Table 2:** Metrics of clinical model

**Model_name**	**Accuracy**	**AUC**	**95% CI**	**Sensitivity**	**Specificity**	**PPV**	**NPV**	**Cohort**
LR	0.905	0.929	0.871–0.986	0.931	0.829	0.941	0.806	train
LR	0.767	0.741	0.597–0.884	0.800	0.667	0.878	0.526	val
LR	0.776	0.868	0.758–0.978	0.750	1.000	1.000	0.312	test
SVM	0.883	0.965	0.934–0.995	0.863	0.943	0.978	0.702	train
SVM	0.700	0.794	0.672–0.916	0.644	0.867	0.935	0.448	val
SVM	0.796	0.855	0.750–0.959	0.773	1.000	1.000	0.333	test
LightGBM	0.891	0.938	0.892–0.984	0.912	0.829	0.939	0.763	train
LightGBM	0.650	0.830	0.726–0.933	0.533	1.000	1.000	0.417	val
LightGBM	0.571	0.820	0.693–0.947	0.523	1.000	1.000	0.192	test

PPV: Positive predictive value; CI: Confidence interval; LR: Logistic regression.

**Figure S2.**
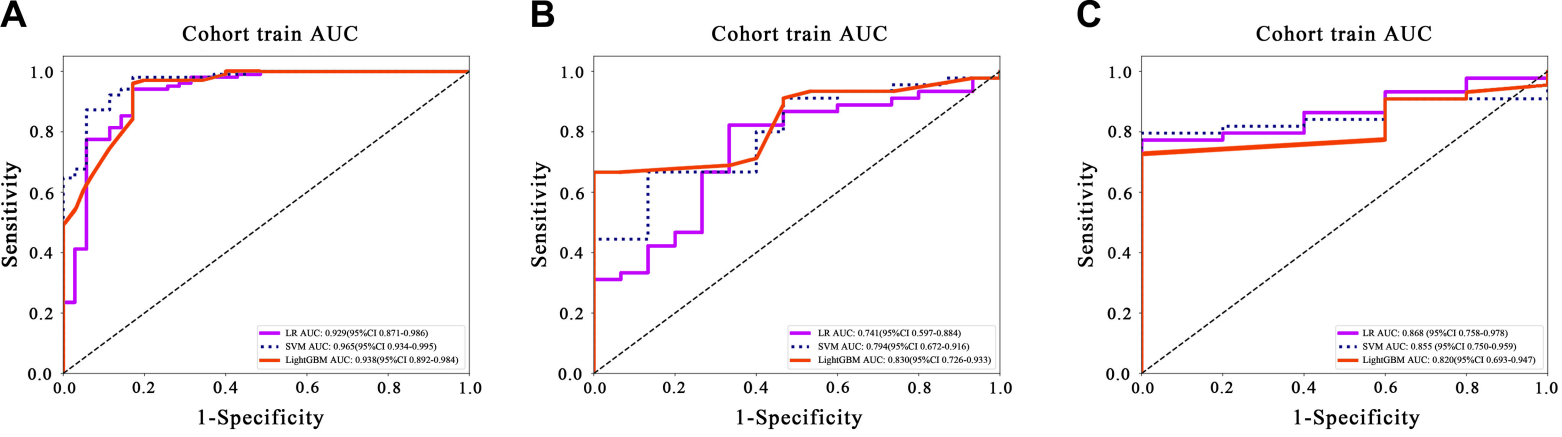
Illustration of the ROC curves for clinical model on different datasets: (A) Training set; (B) Validation set; (C) test set. ROC: Receiver operating characteristic.

The LightGBM model exhibited the highest AUC of 0.820 in the test set (Table 2). This performance highlights its capability to differentiate between the classes, marking its importance in evaluating binary classification models in medical diagnostics (Figure S2).

### Rad signature

**Figure 3.**
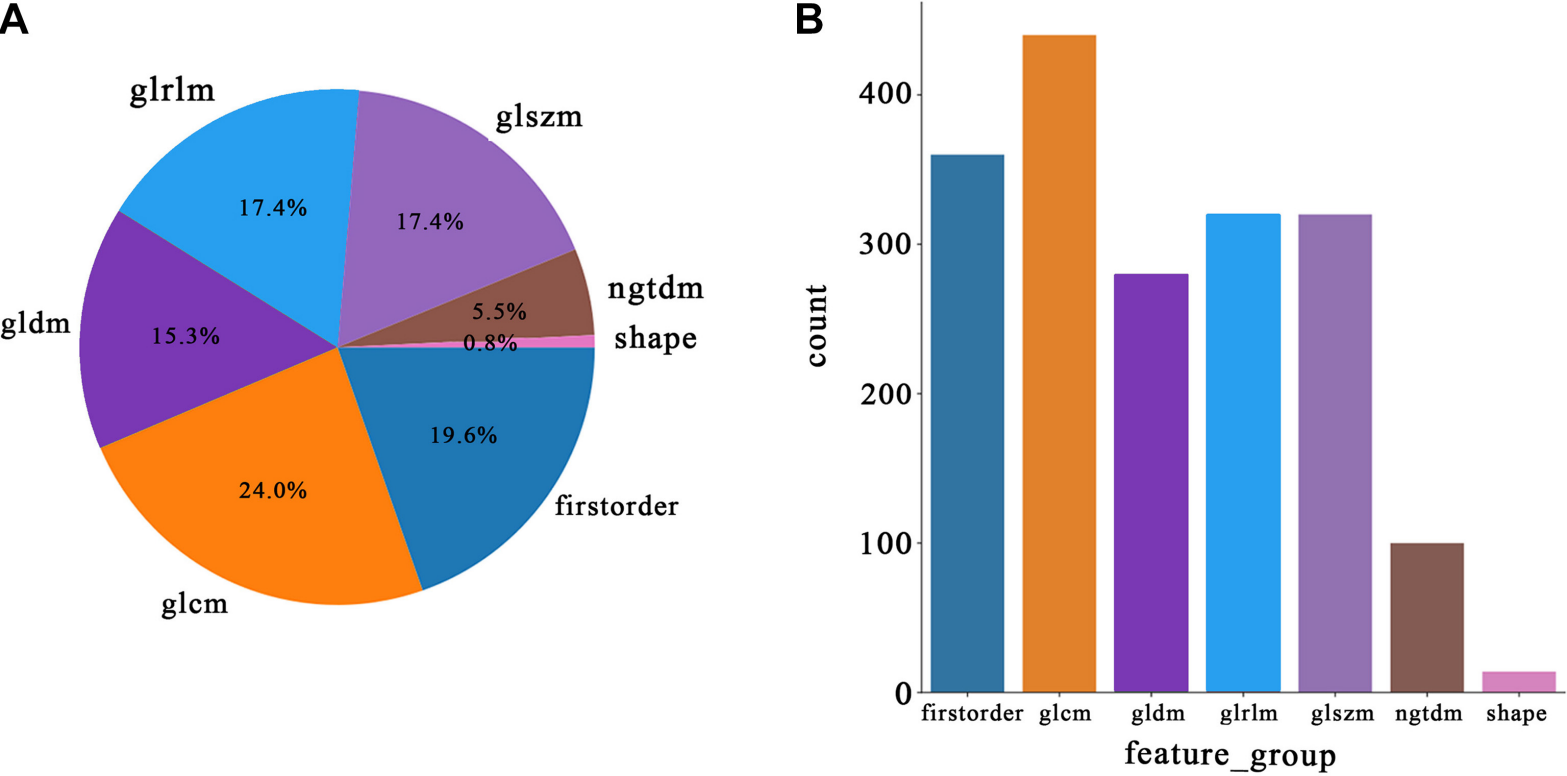
**The distribution of radiomic features: (A) Pie chart: Number of handcrafted radiomic features; (B) Bar chart: Ratio of handcrafted radiomic features.** GLCM: Gray-level co-occurrence matrix; GLRLM: Gray-level run-length matrix; GLSZM: Gray-level size zone matrix; NGTDM: Neighborhood gray-tone difference matrix.

In this study, we compiled a comprehensive dataset of 1,834 handcrafted radiomic features, organized into three primary categories: shape, first-order, and texture. This dataset includes 360 first-order metrics, 14 shape descriptors, and a broad array of texture characteristics. These features were extracted using a specialized program created with Pyradiomics, as detailed at http://pyradiomics.readthedocs.io. The distribution of handcrafted features across the different categories is illustrated in Figure 3.

**Figure 4.**
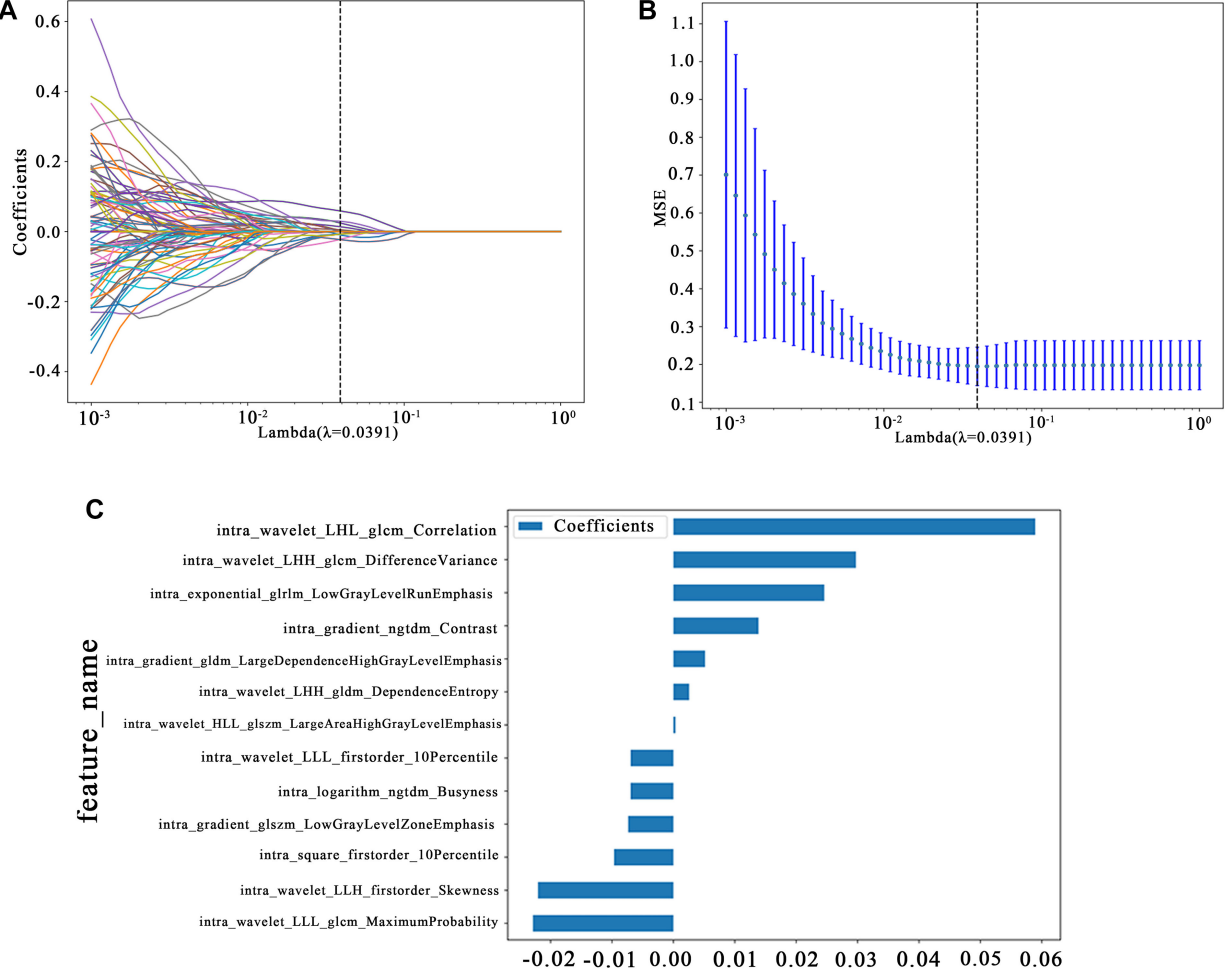
**Dimensionality reduction and selection of radiomic features.** They showcase the coefficients obtained through Least Absolute Shrinkage and Selection Operator regression using 10-fold cross-validation: (A) LASSO regularization path plot; (B) MSE cross-validation error plot; (C) Feature coefficient bar chart. MSE: Mean squared error.

Radiomics Feature Selection: We employed the LassoCV methodology, integrating it with a rigorous 10-fold cross-validation framework to select salient radiomic features. The intricate details of this feature selection process are vividly depicted in Figure 1, offering a comprehensive visual representation of our approach. Figure 4 showcases the coefficients obtained through Lasso regression using 10-fold cross-validation, a technique we utilized in both our Radiomics Signature and INTRA Signature analyses. The left and right sub-figures display the Lasso regularization paths, mean squared error (MSE) values, and relevant radiomic feature weights.

### Metrics

**Figure 5.**
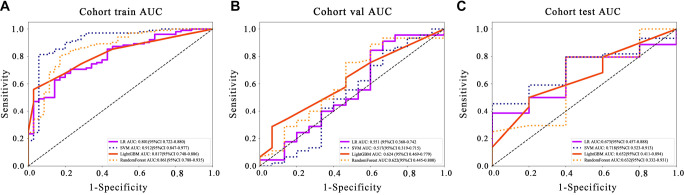
Comparison of ROC curves for the prediction model of early response to ICI based on radiomic features and four machine learning algorithms in 3 datasets: (A) Training set; (B) Validation set; (C) Test set.

Table 3 shows that LightGBM achieves the highest AUC score on the validation set, with a value of 0.624. While this score does not drastically outperform the competition, it surpasses LR and RandomForest, which scored 0.551 and 0.622, respectively. This result indicates that the LightGBM model, being non-linear, is better equipped to capture and generalize the complex relationships within the dataset compared to linear models like LR (see Figure 5).

**Table 3:** Rad signature results

**Model_name**	**Accuracy**	**AUC**	**95% CI**	**Sensitivity**	**Specificity**	**PPV**	**NPV**	**Cohort**
LR	0.679	0.801	0.722–0.880	0.618	0.857	0.926	0.435	train
LR	0.717	0.551	0.360–0.742	0.822	0.400	0.804	0.429	val
LR	0.755	0.673	0.457–0.888	0.773	0.600	0.944	0.231	test
SVM	0.839	0.912	0.847–0.977	0.804	0.943	0.976	0.623	train
SVM	0.700	0.517	0.319–0.715	0.822	0.333	0.787	0.385	val
SVM	0.490	0.718	0.523–0.913	0.432	1.000	1.000	0.167	test
LightGBM	0.591	0.817	0.748–0.886	0.461	0.971	0.979	0.382	train
LightGBM	0.333	0.624	0.469–0.779	0.133	0.933	0.857	0.264	val
LightGBM	0.469	0.652	0.411–0.894	0.432	0.800	0.950	0.138	test
RandomForest	0.803	0.861	0.788–0.935	0.794	0.829	0.931	0.580	train
RandomForest	0.683	0.622	0.445–0.800	0.733	0.533	0.825	0.400	val
RandomForest	0.755	0.632	0.332–0.931	0.773	0.600	0.944	0.231	test

PPV: Positive predictive value; CI: Confidence interval; LR: Logistic regression.

The higher AUC value for LightGBM highlights its improved ability to distinguish between positive and negative classes across diverse and complex scenarios. This supports the notion that non-linear models, due to their capacity to model intricate interactions and non-linear dependencies, are often more effective for tasks where relationships between features are not straightforward, thus providing a more robust fit to the data.

### Deep learning radiomics signature

#### Results

**Figure S3.**
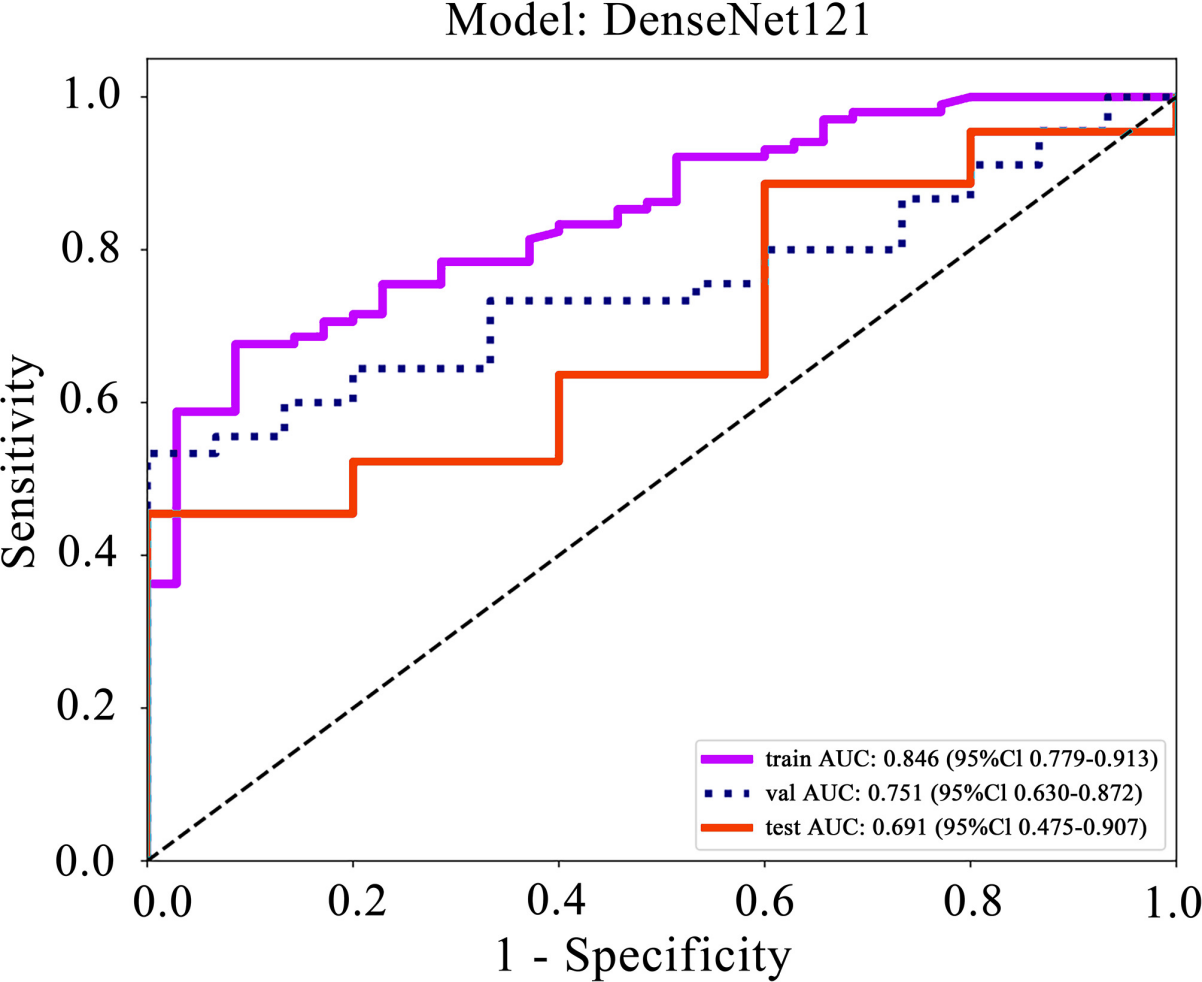
Illustration of the ROC curves for Deep Learning Signature on different datasets: (A) Training set; (B) Validation set; (C) Test set. ROC: Receiver operating characteristic.

The performance of the DenseNet121 model, as indicated in the provided data (Table 4), shows promising results in terms of its ability to discriminate between classes, particularly highlighted by its AUC scores across different cohorts (Figure S3).

**Table 4:** Metric results for deep learning radiomics signature

**Model_name**	**Accuracy**	**AUC**	**95% CI**	**Sensitivity**	**Specificity**	**PPV**	**NPV**	**Cohort**
Densenet121	0.730	0.846	0.7793–0.9126	0.667	0.914	0.958	0.485	train
Densenet121	0.633	0.751	0.6300–0.8722	0.511	1.000	1.000	0.405	val
Densenet121	0.490	0.691	0.4747–0.9071	0.432	1.000	1.000	0.167	test

PPV: Positive predictive value; CI: Confidence interval.

**Figure 6.**
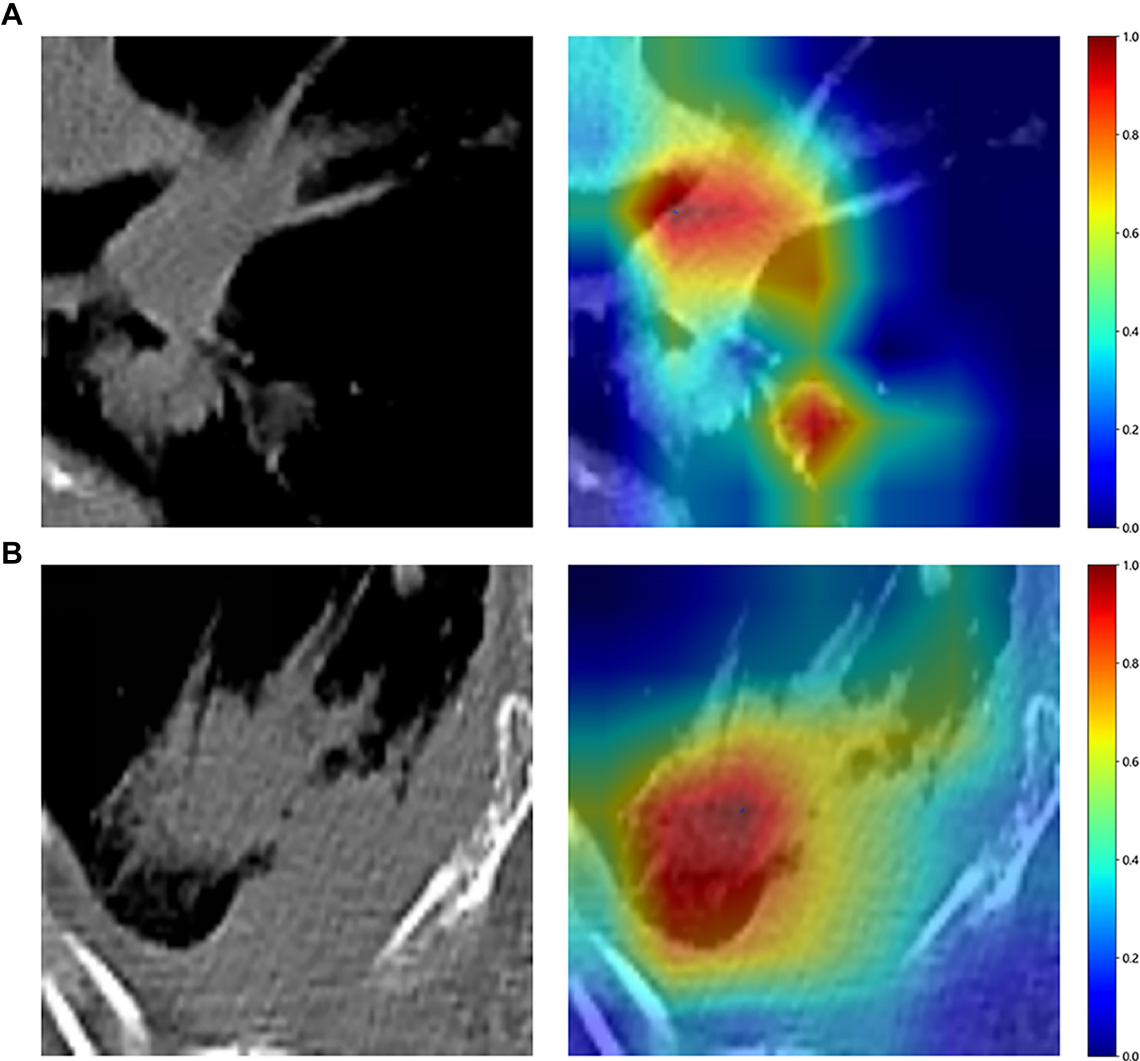
**Visual analysis of heat map based on Grad-CAM technology.** They present the gradient-weighted class activation mapping visualizations for two typical samples: (A) Identified as “689166”; (B) Identified as “866891”. These visualizations are instrumental in demonstrating how the model focuses on different regions of the images for making its predictions. Among them, red highlights the area with the largest contribution, and blue represents the area with the smallest contribution.

- Training cohort: The DenseNet121 model attained an AUC of 0.846, with a 95% confidence interval (CI) between 0.7793 and 0.9126. This high AUC indicates strong discriminative capability during the training phase.
- Validation cohort: In the validation set, the model achieved an AUC of 0.751, with a CI from 0.6300–0.8722, still reflecting a relatively high predictive accuracy.
- Test cohort: On the test dataset, the AUC measured 0.691, with a CI ranging from 0.4747–0.9071. Although lower than the training and validation phases, this score still suggests a moderate ability to distinguish between the positive and negative outcomes.

In all phases, especially notable are the high specificity and positive predictive value (PPV) scores, reaching 1.000 in both the validation and test cohorts. This result illustrates that when the model forecasts a positive class, it is notably accurate, with no false positives documented. However, the sensitivity scores are comparatively lower, suggesting that while the model is excellent at confirming cases when present, it misses a significant number of positive cases (low true positive rate).

When contrasting these results with the radiomics signature, the DenseNet121 deep learning approach potentially offers an improvement due to its capability to automatically learn and generalize from intricate image features across multiple levels of abstraction. This capability often translates into a more nuanced understanding and exploitation of the underlying patterns in medical images compared to more conventional radiomic approaches, which rely on predefined features. Thus, DenseNet121’s performance, particularly in terms of its high specificity and PPV in the test cohort, underscores its potential for more accurate and reliable clinical applications, although there might be room for improvement in its sensitivity to ensure fewer positive cases are missed.

#### Gradient-weighted Class Activation Mapping (Grad-CAM) visualization

To probe the deep learning models’ recognition capabilities across different samples, we employed the Grad-CAM technique for visualization. In the implementation of Grad-CAM, we focused on the analysis of the last convolutional layer feature map of DenseNet121 and used it to generate a heat map that reflects the metabolically active region at the edge of the tumor. Due to the dense connection mechanism of DenseNet, deep features can retain fine-grained semantic information through cross-layer aggregation. Experiments show that the convolutional layer at the end of the last dense block of DenseNet121 contributes the most to the final classification decision. After the global average pooling of the high-dimensional feature map output by this layer, the channel gradient weight directly reflects the degree of attention of the model to the tumor area. Through Grad-CAM, we further localized the image areas associated with lung malignant tumors (such as irregularly enhanced areas on the edges and peripheral edema zones), while the activation areas of benign tumors were concentrated in the internal uniform texture areas. This analysis verifies the interpretability of the model decision, and the relevant heat map comparison will be presented in Figure 6 of the results section.

**Figure 7.**
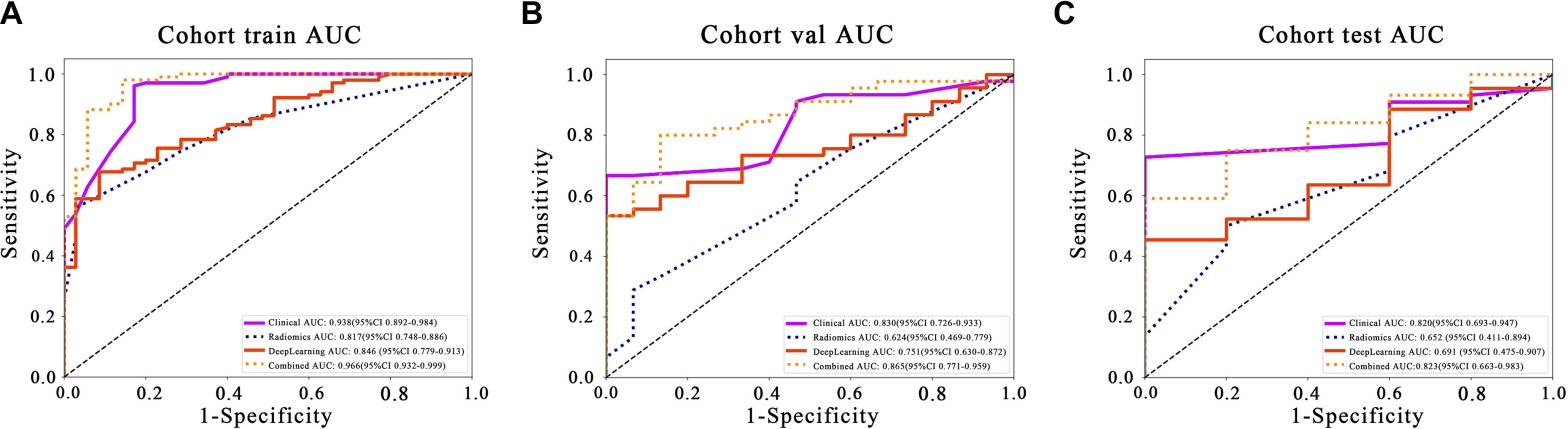
**Comparison of ROC curves for the single signature model and combined model of early response prediction to ICI in 3 datasets: (A) Training set; (B) Validation set; (C) Test set.** The combined model consistently outperforms the single signature model across training, validation, and test cohorts, demonstrating the efficacy of integrating multi-modal data and analytical approaches.

**Figure 8.**
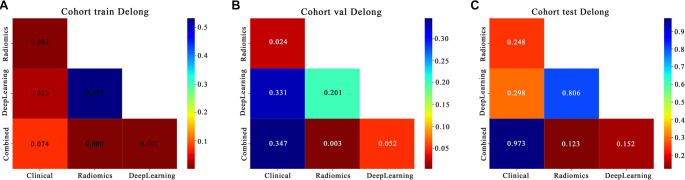
**Results of DeLong test performed separately for the single signature model and combined model of early response prediction to ICI in 3 datasets: (A) Training set; (B) Validation set; (C) Test set.** The results show that the AUC values of combined model compared with radiomics and DeepLearning model in the training set showed statistically significant differences (*P* < 0.05), indicating that the performance of combined model was significantly better than that of Radiomics and DeepLearning model. In the validation set, the AUC values of combined model compared with radiomics model show statistically significant differences (*P* < 0.05).

### Clinical use

Analyzing the AUC scores across different models and cohorts (Table 5), the Combined model consistently demonstrates an improvement over the single signature model (Figure 7). This trend is evident in training, validation, and test cohorts, underscoring the efficacy of integrating multiple types of data or analytical approaches.

**Table 5:** Metrics on different signature

**Signature**	**Accuracy**	**AUC**	**95% CI**	**Sensitivity**	**Specificity**	**PPV**	**NPV**	**Cohort**	
Clinical	0.891	0.938	0.8924–0.9840	0.912	0.829	0.939	0.763	train	
Radiomics	0.591	0.817	0.7478–0.8861	0.461	0.971	0.979	0.382	train	
DeepLearning	0.730	0.846	0.7793–0.9126	0.667	0.914	0.958	0.485	train	
Combined	0.942	0.966	0.9322–0.9995	0.971	0.857	0.952	0.909	train	
Clinical	0.650	0.830	0.7258–0.9334	0.533	1.000	1.000	0.417	val	
Radiomics	0.333	0.624	0.4687–0.7787	0.133	0.933	0.857	0.264	val	
DeepLearning	0.633	0.751	0.6300–0.8722	0.511	1.000	1.000	0.405	val	
Combined	0.800	0.865	0.7709–0.9595	0.778	0.867	0.946	0.565	val	
Clinical	0.571	0.820	0.6934–0.9475	0.523	1.000	1.000	0.192	test	
Radiomics	0.469	0.652	0.4109–0.8937	0.432	0.800	0.950	0.138	test	
DeepLearning	0.490	0.691	0.4747–0.9071	0.432	1.000	1.000	0.167	test	
Combined	0.612	0.823	0.6627–0.9827	0.568	1.000	1.000	0.208	test	

PPV: Positive predictive value; CI: Confidence interval.

**Figure S4.**
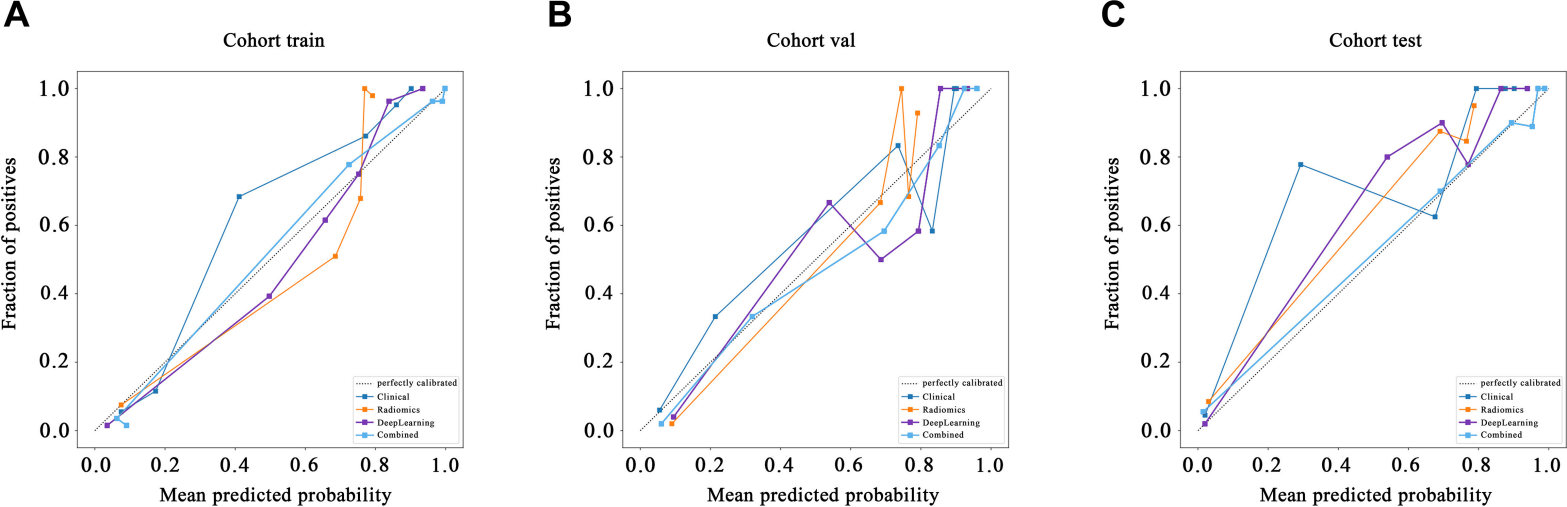
Calibration curve on different datasets: (A) Training set; (B) Validation set; (C) Test set.

Calibration Curve: The HL test plays a crucial role in evaluating the calibration of a predictive model by comparing the predicted probabilities with the actual outcomes. Higher HL *P* values indicate better calibration, reflecting closer alignment between the model’s predictions and observed outcomes. In our study, the Combined model exhibited outstanding calibration, as evidenced by HL test statistics of 0.964 in the training cohort, 0.633 in the validation cohort, and 0.140 in the test cohort. These results highlight the model’s high effectiveness in accurately mirroring observed data (Figure S4).

DeLong Test: The DeLong test is a method for comparing whether there is a significant difference in the AUC of two or more models. In other words, it helps us to judge whether a model is significantly better than another model. If the *P* value is less than 0.05, one of the models is significantly better than the other. While the DeLong test confirmed no significant AUC difference between the Deep Learning model and Clinical model in the external test cohort (*P* ═ 0.298; AUC 95% CI overlap: Deep Learning model [0.475–0.907] vs. Clinical model [0.693–0.947]), the Combined model demonstrated superior overall performance in both the training and validation cohorts, as shown in Figure 8.

**Figure 9.**
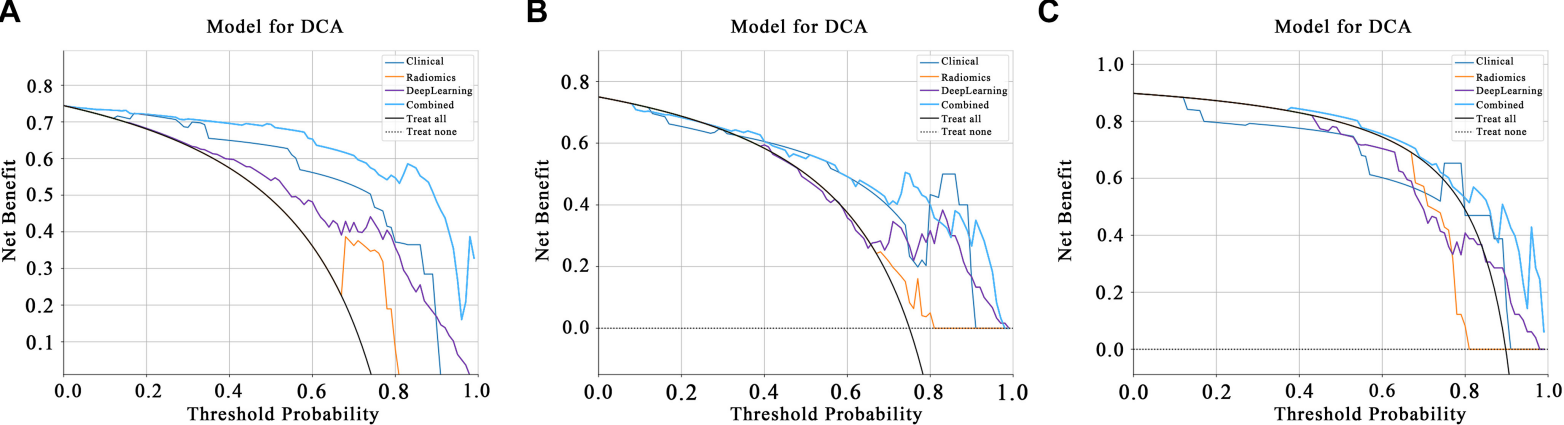
**Comparison of DCA curves of prediction models (Clinical, Radiomics, DeepLearning, Combined model) in different datasets: (A) Training set; (B) Validation set; (C) Test set.** The results show that in 3 datasets, the DCA curve of the combined model is higher than other models in most of the risk threshold intervals, that is, in most of the threshold ranges, the decision made by the combined model can bring higher net benefits. DCA: Decision curve analysis.

DCA: Figure 9 presents the DCA for the training and testing sets. These curves demonstrate that our fusion model offers significant advantages in terms of its predictive probabilities.

**Figure 10.**
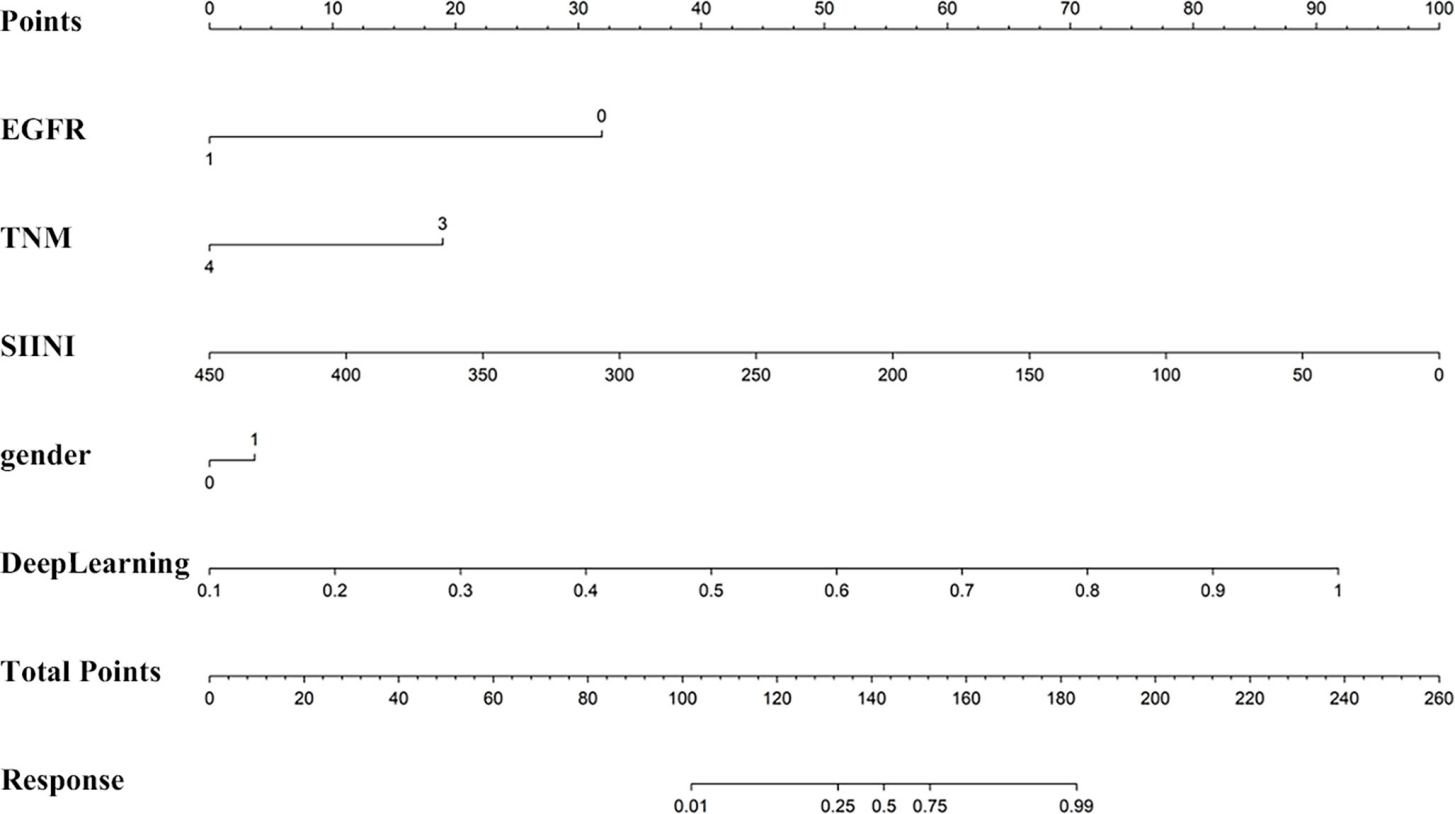
**Nomogram constructed based on the EGFR, TNM, SIINI, gender, and DeepLearning model.** It show that EGFR mutation-negative status, TNM stage III, low SIINI score, male and elevated deep learning index collectively predict a heightened likelihood of early response to ICIs.

Nomogram: Figure 10 Nomograms suggest that EGFR, TNM, SIINI, gender, and Deep Learning are incorporated into the combined model, and the corresponding scores can predict the unresectable NSCLC response to ICI treatment in the early stage. Therefore, EGFR mutation negative, TNM stage III, low SIINI score, and high deep learning index indicate a greater tendency to have an early response to ICIs.

## Discussion

The persistent global burden of lung cancer, characterized by high incidence and mortality rates, has driven multidisciplinary efforts to identify clinically actionable biomarkers for predicting ICI response in unresectable NSCLC. Current biomarker discovery paradigms span traditional histopathological evaluation to molecular profiling [29–31]. Prior studies have identified numerous hematological parameters as prospective prognostic markers, including PD-L1, TMB, the NLR, dNLR, the PLR, the PNI, the SII, the ALI, alongside hemoglobin concentrations, among others [15, 32–34]. These parameters reflect distinct aspects of the tumor-host interface, yet their clinical application remains constrained by high costs, inherent biological variability, and limited capacity to capture the complex multidimensional nature of antitumor immunity.

Throughout immunotherapy for NSCLC, lymphocytes play an instrumental role in tumor defense by inducing apoptosis and inhibiting tumor cell proliferation and migration [33]. The reduction in lymphocytes may reflect a decrease in CD4+ T lymphocytes, leading to a weakened lymphocyte-mediated immune response to malignant tumors [35]. Furthermore, a study by Lee et al. [36] suggests a possible link between serum hemoglobin levels and outcomes in lung cancer patients. Additionally, some studies indicate that NSCLC patients with lower baseline platelet (PLT) and NLR levels tend to have better prognoses [34]. These findings suggest that the tumor inflammatory microenvironment may be closely related to anti-tumor immune responses, which can significantly impact the prognosis of NSCLC patients [32, 37, 38]. Therefore, our study identifies a novel indicator (SIINI), which considers various aspects of the body, offering a more comprehensive evaluation of immune, inflammatory, and nutritional indicators. The SIINI integrates neutrophil count, lymphocyte count, platelet count, hemoglobin level, serum albumin level, and BMI, offering a comprehensive evaluation of the nutritional, inflammatory, and immune status in patients with NSCLC. SIINI can be used not only to predict patient prognosis but also to assess treatment efficacy and potentially offers greater clinical significance compared to established indicators such as NLR, PLR, PNI, SII, and ALI.

Furthermore, within this landscape, CT-based radiological biomarkers hold unique translational potential due to their intrinsic non-invasive nature and universal acquisition during standard diagnostic workflows [39]. Unlike invasive tissue sampling techniques, which are limited by spatial sampling bias, advanced imaging modalities enable comprehensive three-dimensional tumor characterization, capturing both intralesional heterogeneity and peritumoral microenvironmental features with millimeter-level spatial resolution [40]. A paramount advantage of deep learning in radiomics feature extraction lies in its adaptability and proficiency in discerning patterns from image data [41, 42]. Presently, it epitomizes the pinnacle of image analysis and categorization, consistently surpassing antecedent image analysis methodologies [43, 44]. Rakaee et al. [45] constructed a machine learning model based on TIL scoring to forecast the response of NSCLC to ICIs. In a parallel vein, Vanguri et al.amalgamated radiological, histopathological, and genomic attributes to gauge the predictive potential of immune therapy responses in NSCLC. Through the application of machine learning, they consolidated multimodal attributes into a risk prediction paradigm. The investigation revealed that the multimodal framework attained an AUC of 0.80, surpassing any solitary variable. These discoveries provide a quantitative foundation for harnessing multimodal integrated attributes in conjunction with machine learning to augment the precision of anticipating immune therapy responses in NSCLC patients [46]. In our research, the DenseNet121 model manages to categorize responses at each evaluation point, essentially augmenting the training dataset, even with a small sample size. Furthermore, the validation set proves to be an unexpectedly good predictor in distinguishing responders from non-responders. Moreover, external validation demonstrated good generalizability (AUC ═ 0.823), confirming the universality of the core predictive factors.

This study primarily focused on patients with unresectable NSCLC at advanced TNM stages. For patients with EGFR-positive NSCLC, targeted therapy remains the preferred treatment approach. However, the emergence of resistance to targeted therapy presents an inevitable challenge. In this context, ICIs have emerged as a promising therapeutic option for patients with unresectable NSCLC. This study suggests that EGFR mutation status is associated with the response to ICIs, with EGFR-negative patients more likely to exhibit an early response to ICI treatment, consistent with the findings of Jiang and colleagues [47, 48]. The SIINI serves as both a clinical efficacy biomarker and a prognostic indicator, offering distinct advantages in accessibility, safety, cost efficiency, reproducibility, and adaptability for longitudinal monitoring. These strengths arise from its calculation using routine clinical parameters, including complete blood count, biochemical profiles, and BMI. However, some inflammatory components in SIINI are susceptible to various confounding factors, which may lead to differences in model performance across different data sets and introduce bias into the research results. To mitigate these limitations, integrating SIINI with complementary inflammatory biomarkers and adopting longitudinal assessments could effectively enhance diagnostic accuracy and reduce measurement variability.

Although the results are promising, this research faced several constraints. Initially, the number of participants was limited. While data from two medical centers in China were included, further validation through larger prospective studies is needed. Additionally, real-world data collection reveals challenges in obtaining indicators such as TMB and circulating tumor DNA (ctDNA) [49], which may be due to high detection costs or inconsistent detection standards in local medical centers. Future investigations should synergistically integrate multi-omics biomarker panels, expand prospective multicenter validation frameworks, and achieve statistically powered cohort sizes (*n* ≥ 400), collectively addressing the current limitations in predictive robustness and the scalability of clinical implementation.

## Conclusion

This study examined the early predictive capability of a CT-based deep learning model, combined with the inflammation parameter SIINI, to forecast the response of unresectable NSCLC patients to ICIs. By facilitating the selection of appropriate candidates for ICI treatment, this research seeks to minimize unnecessary financial and time burdens on patients while offering a viable approach to precision therapy.

## Supplemental data

**Table S1:** Univariable and multivariable analysis of clinical features

**Feature_name**	**OR**	**OR lower 95% CI**	**OR upper 95% CI**	***P* value**	**OR**	**OR lower 95% CI**	**OR upper 95% CI**
EGFR	0.560	0.505	0.621	<0.05	0.632	0.571	0.7
TNM	0.775	0.679	0.884	<0.05	0.873	0.784	0.971
Modality	0.777	0.538	1.123	0.258			
ECOG	0.881	0.811	0.957	<0.05	0.967	0.906	1.033
NLR	0.921	0.891	0.951	<0.05	0.982	0.949	1.016
pathological_type	0.925	0.817	1.047	0.302			
treatment_lines	0.960	0.875	1.055	0.478			
Smoking	0.969	0.853	1.100	0.677			
BMI	0.996	0.980	1.012	0.706			
Type	0.996	0.959	1.034	0.847			
SIINI	0.998	0.997	0.998	<0.05	0.999	0.998	1.0
PLR	1.000	0.999	1.000	0.569			
ALI	1.000	1.000	1.000	0.542			
SII	1.000	1.000	1.000	0.275			
Age	1.004	0.997	1.011	0.347			
PNI	1.007	0.996	1.018	0.272			
medication_regimen	1.027	0.916	1.151	0.704			
basic_disease	1.097	0.966	1.247	0.232			
PD_L1	1.104	1.018	1.197	<0.05	1.038	0.977	1.103
Gender	1.298	1.096	1.536	<0.05	1.17	1.03	1.328

ECOG: Eastern Cooperative Oncology Group; BMI: Body mass index; SIINI: Systemic immune-inflammatory-nutritional index.

**Table S2:** Sensitivity analysis of clinical features

	**Feature**	**AUC**	**Sensitivity**	**Specificity**
1	Gender	0.707	0.842	0.571
2	EGFR	0.791	0.868	0.714
3	pathological_type	0.633	0.553	0.714
4	TNM	0.6	0.342	0.857
5	SIINI	0.842	0.684	0.857
